# Nodular and Micronodular Basal Cell Carcinoma Subtypes Are Different Tumors Based on Their Morphological Architecture and Their Interaction with the Surrounding Stroma

**DOI:** 10.3390/diagnostics12071636

**Published:** 2022-07-05

**Authors:** Mircea-Sebastian Șerbănescu, Raluca Maria Bungărdean, Carmen Georgiu, Maria Crișan

**Affiliations:** 1 Department of Medical Informatics and Biostatistics, University of Medicine and Pharmacy of Craiova, 200349 Craiova, Romania; mircea_serbanescu@yahoo.com; 2 Department of Pathology, Iuliu Haţieganu University of Medicine and Pharmacy, 400012 Cluj-Napoca, Romania; carmen.georgiu@elearn.umfcluj.ro; 3 Department of Histology, Iuliu Haţieganu University of Medicine and Pharmacy, 400012 Cluj-Napoca, Romania; maria.crisan@elearn.umfcluj.ro

**Keywords:** basal cell carcinoma, Haralick texture features, histogram moments, semantic segmentation, peritumoral cleft

## Abstract

Basal cell carcinoma (BCC) is the most frequent cancer of the skin and comprises low-risk and high-risk subtypes. We selected a low-risk subtype, namely, nodular (N), and a high-risk subtype, namely, micronodular (MN), with the aim to identify differences between them using a classical morphometric approach through a gray-level co-occurrence matrix and histogram analysis, as well as an approach based on deep learning semantic segmentation. From whole-slide images, pathologists selected 216 N and 201 MN BCC images. The two groups were then manually segmented and compared based on four morphological areas: center of the BCC islands (tumor, T), peripheral palisading of the BCC islands (touching tumor, TT), peritumoral cleft (PC) and surrounding stroma (S). We found that the TT pattern varied the least, while the PC pattern varied the most between the two subtypes. The combination of two distinct analysis approaches yielded fresh insights into the characterization of BCC, and thus, we were able to describe two different morphological patterns for the T component of the two subtypes.

## 1. Introduction

Basal cell carcinoma (BCC) is the most frequent type of skin cancer in humans [1,2]. Histopathology is considered the “gold standard” in the diagnosis of oncological skin pathology [3,4,5]. The origin of BCC cells is currently believed to be the basal cells located in the interfollicular epidermis or the follicular bulges [5,6]. Although BCC histology has a wide range of morphological characteristics, constant features are the islands and nests with peripheral palisading basaloid cells with scant cytoplasm and hyperchromatic nuclei, often with stromal retraction and fibromyxoid stroma [6].

The latest version of the WHO Classification of Skin Tumors recognizes 10 different subtypes of BCC and divides them into lower- and higher-risk groups based on recurrence [6]. However, some studies showed poor inter-observer reproducibility when classifying these subtypes, emphasizing the practical challenges pathologists face in everyday practice when using the present criteria [7,8]. A study by Nedved et al. showed fair agreement (k 0.301, *p* < 0.001) in BCC subtyping by six dermatopathologists, but substantial agreement (k 0.699, *p* < 0.001) in dividing them into low- and high-risk groups [8].

Pathologists usually report BCC by subtype, and afterward, clinicians decide the patient management [8]. Thus, in the context of significant inter-observer reproducibility amongst pathologists [7,8] and frequent admixture of multiple subtypes within a single tumor [9,10], confusion may arise.

However, according to some authors, the most problematic definition is that of the micronodular subtype because the definition does not take into consideration the tangential sections of other subtypes or irregularity near the margin of large nodules, which can also mimic micronodules [11,12]. Moreover, because the previous definition of micronodules states that they should be smaller than 0.15 mm but did not offer a minimal number of those small nodules in order for the tumor to be classified as micronodular, some authors implied that the presence of a single solitary micronodule in a typical nodular subtype can warrant classification as micronodular BCC [7] or a mixed-type tumor. However, the definition of the micronodular subtype improved in the fourth edition of the WHO Classification of Skin Tumors, where alongside the required size of micronodules (less than 0.15 mm in diameter), it also states that they should make up to more than 50% of the tumor [13].

This study focused on the most common low-risk subtype, namely, nodular BCC, and a high-risk subtype, namely, micronodular BCC. Deep learning showed promising results in image comprehension, reconstruction and reasoning, and particular convolutional neural network techniques were widely used for classification and segmentation tasks across a wide range of applications [14,15,16], allowing for better visualization of histology images, sometimes finer than the human eye [17]; therefore, digital pathology has advantages in terms of time savings and performance [18] and can improve diagnostic efficiency and accuracy [19].

Various aspects were studied in BCC diagnosis via deep learning methods using several network architectures as follows. By using U-Net for pixel segmentation and a proprietary algorithm for classification, van Zon et al. aimed to distinguish normal tissue from BCC for margin control in Mohs surgery and obtained an area under the curve (AUC) of 90% [20]. Campanella et al. also distinguished between normal and BCC tissue using a different deep learning convolutional network architecture, namely, ResNet34, and obtained high sensitivity, correctly identifying all sections with BCC and a lower specificity of 94% with four false-positive results [21]. Using GoogLeNet Inception v3, Jiang et al. studied BCC detection from images captured using using smartphones from microscope ocular lens and attained a similar performance to those captured from slides and even from whole-slide images [22]. Using the VGG11 network architecture, Kimeswenger et al. offered an artificial neural network that can identify and classify BCC on whole-slide images by comparing the network results with the eye movements of pathologists and concluded that software can improve the diagnostic quality of the human eye [23]. They also recognized BCC with a specificity and sensitivity of 95% [23]. In a previous study, the present group of authors designed a deep learning convolution-based software using transfer learning from three general-purpose image classification networks: AlexNet, GoogLeNet and ResNet-18. This software was able to classify subtypes of BCC, such as superficial, nodular (with adenoid, nodulo-cystic and keratotic variants), pigmented, with adnexal differentiation, micronodular and infiltrating [24].

To our knowledge, there are no studies in the literature that compared nodular and micronodular subtypes of BCC using deep learning techniques.

The two subtypes can be characterized using histological aspects, such as the tumor stroma, stromal retraction, peripheral palisading cells and tumor island without palisading cells. Therefore, in the following paragraphs, we consider the current literature on these aspects.

### 1.1. Tumor Stroma

In the process of tumorigenesis, not only cancer cells play an essential role, but also the tumor microenvironment [25], thus creating a habitat that protects the tumor from the immune system [26].

The vast majority of BCC have a fibro-myxoid stroma [6], which is composed of glycosaminoglycan-based ground substance with a complex network of collagen, elastin and fibronectin [27], along with inflammatory cells and fibroblasts that interact with tumor cells via growth factors or extracellular matrix proteins secretion, thus influencing tumor growth and progression, as well as angiogenesis or metastasis [28,29,30]. In the inflammatory infiltrate of BCC, stroma lymphocytes are dominant, having both a pro- and an anti-tumoral effect, though the anti-tumoral effect prevails, where some studies showed an increase in IL-4 and IL-10 Th2 cytokines in BCC stroma involved in tumor proliferation [31,32]. Fibroblasts present in the stroma have a particular phenotype and markers [33], thus participating in the promotion of tumor growth [34] and progression [33,35] through the production of cytokines and extracellular matrix components [34]; the presence of these fibroblasts was demonstrated in both the tumor and peritumoral stroma of BCC [36].

Nonetheless, when the high-risk and low-risk subtypes were compared, changes in the stroma component were observed. Immunoreactivity to beta-catenin, which is a protein involved in the expression of membrane type-matrix 1 metalloproteinase (MT1-MMP) [37,38], is increased at the invasion front of the micronodular versus nodular subtype [39]. In terms of the amount of inflammatory infiltrate, high-risk subtypes were found to have a more abundant infiltrate [40]. Furthermore, Th1 and Th2 are more abundant in high-risk subtypes [41].

Although the functional role of the peritumoral stroma is not clearly elucidated in BCC, it was observed that there are qualitative and quantitative differences between subtypes [28,42]. In a comparison between micronodular and nodular subtypes, a difference in the presence of actin was found. Actin was present in most cases of the micronodular subtype and was absent in the nodular subtype [43], which could explain the aggressiveness of the invasion of the micronodular subtype compared with the nodular one since the microfilaments responsible for cell motility are mainly composed of actin [44]. In terms of the histological appearance of the stroma, high-risk subtypes show more intense hyalinization, while a more fibrous stroma is associated with low-risk subtypes of BCC [45,46]. In the micronodular subtype, some researchers report a loss of stromal response [47], while others show the presence of a fibromyxoid stroma [40]. Therefore, there are some different opinions in the literature on the micronodular stroma subtype, which we aimed to study using different methods than those studied so far, namely, by using artificial intelligence.

### 1.2. Tumor Island (Including Peripherally Palisaded Basaloid Cells)

According to an X-chromosome inactivation study, BCC is a monoclonal neoplastic development of basaloid epithelial cells embedded in a polyclonal connective tissue stroma [48]. As previously mentioned, BCCs originate in the basal cells of the interfollicular epidermis or follicular bulges [5,6], and therefore, will have properties specific to this origin, such as cell adhesion specific to epithelial tissues. Although matrix metalloproteinases (MMPs) are involved in modulating the tumor microenvironment, they are also engaged in activating cell adhesion molecules [49], one of which is E-cadherin [50,51], which is essential in the cell-to-cell adhesion of epithelial tissues. Moreover, together with beta-catenin, it creates a protein complex that is involved in the mesenchymal–epithelial transition, and thus, the two are directly involved in tumor progression [52]. The presence of beta-catenin, especially in the membrane of tumor cells of the micronodular subtype of BCC, suggests another mechanism involved in this subtype [53].

Although there are common characteristics of all BCC subtypes, such as originating from the same cell type, these subtypes have different histological morphologies and biological behaviors [54,55]. Low-risk BCC types have a slow and indolent growth pattern with high bcl-2 protein labeling, while those with an aggressive subtype, either mixed or pure, display heterogeneous bcl-2 labeling [56].

Given the differences in bcl-2 protein expression, beta-catenin and MMP-1 expression in tumor islands between the nodular and micronodular subtypes, we believe that an evaluation via deep learning methods using transfer learning could provide additional information that is not visible to the human eye or available using immunohistochemical staining.

### 1.3. Peritumoral Cleft

The presence of peritumoral clefts or retraction spaces at the periphery of BCC tumor islands is frequent and can be a diagnostic clue when present [6,57]. The exact mechanism by which these peritumoral clefts form remains unknown; however, various hypotheses were proposed. Although in the past, it was stipulated that these retractions are actually a processing artifact due to fixation and dehydration [57], this was refuted by the studies of several authors that demonstrated the involvement of the tumor microenvironment. Levin et al. and Ghita et al. demonstrated the presence of in vivo peritumoral clefts using reflectance confocal microscopy. Ghita et al. observed dark spaces that surrounded tumor islets [58,59]. These findings were corroborated by Ulrich et al.’s research, which went even further to state that in the peritumoral clefts, there are mucin deposits [60]. Another study concluded that the origin of these spaces comes from the extracellular matrix degradation that occurs during tumor growth [61].

Another theory is that peritumoral retraction is caused by epithelial membrane disintegration. Some studies demonstrated the lack of laminin-5 in the area surrounding tumor nests and suggested an improper structure or an absence of the hemidesmosome-anchoring filament complex in BCC, which leads to cleavage of the basal membrane [62,63]. Breakdown of the basal membrane was also demonstrated using staining for Ep-CAM and cytokeratins by Rios-Martin et al. [57]. However, not all authors agree with this result, with some suggesting that laminin does not play a substantial part is cleft formation [64].

More recent studies reflected on the effects of MMPs, such as MMP-2 and MMP-9, and stated that extracellular matrix remodeling plays a significant part alongside a decreased expression of adhesion molecules [65], although other metalloproteinases, such as stromelysin-3, were believed to be involved in tumor invasion via degradation of the matrix of the stroma [66]. Some researchers demonstrated an increased expression of MMP-2 in the stroma of high-risk compared to low-risk BCC subtypes, suggesting a role in tumor invasiveness [67]; however, when the presence of MMP-2 and MMP-9 was studied in the peritumoral space, no statistically significant correlations were observed between these and the space [65].

Peritumoral clefts are common in nodular BCC [6], but some studies stated that they are uncommon in the micronodular subtype [68,69,70].

Hence, there are several theories regarding why and how these peritumoral clefts exist and whether they are present or not in the micronodular BCC subtype. These differences of opinion in the literature prompted us to study these spaces using deep learning methods.

Through this study, we aimed to identify the morphological differences that occur in these two subtypes, using, on one hand, the classical morphometric approach with gray-level co-occurrence matrix features and histogram moments, and, on the other, an approach based on deep learning segmentation.

## 2. Materials and Methods

### 2.1. Materials

The dataset included consecutive cases of N (n = 46), MN (n = 12) and mixed (n = 31) subtypes of BCC that were presented at the Cluj-Napoca Clinical Municipal Hospital in Romania between 2019 and 2021.

Prior to data collection, approval from the Research Ethical Committee (approval no. 7749/21 September 2021) was obtained.

The surgically removed tissue was histologically treated, and the slides were stained with standard hematoxylin and eosin staining. All the slides were scanned using the 20× objective of the Pannoramic SCAN II, 3DHISTECH (Budapest, Hungary), resulting in whole-slide images (WSI).

Representative BCC images with 1920 × 1017 pixels in 32-bit RGB (red, green, blue) color space, representing 0.038 square microns per pixel, were extracted from WSIs by pathologists with experience in dermatopathology. From the total of 417 images, 201 images were labeled as the micronodular subtype, while the remaining 216 images were labeled as the nodular subtype. Even in mixed-subtype WSIs, the images that were selected presented one of the specified subtypes exclusively.

### 2.2. Methods

#### 2.2.1. Dataset Preparation

Pathologists labeled images as either nodular (N) or micronodular (MN) based on the definitions from the 4th edition of WHO Classification of Skin Tumors [6]. When assessing the micronodular subtype, the invasive character in the deeper part of the tumor was also taken into consideration.

In our assertion, the BCC interaction with the surrounding tissue creates four distinct morphological components (patterns):

- Tumor (T)—representing the center of the BCC islands, where interactions with the surrounding stroma are limited (Section 1.2);
- Touching tumor (TT)—representing the peripherally palisaded part of the tumor where the interactions with the surrounding stroma are maximal (Section 1.2);
- Peritumoral cleft (PC)—representing the peritumoral clefts where the interactions with the tumor are maximal (Section 1.3);
- Stroma (S)—representing the surrounding stroma where interactions with the tumor are limited (Section 1.1).

**Figure 1:**
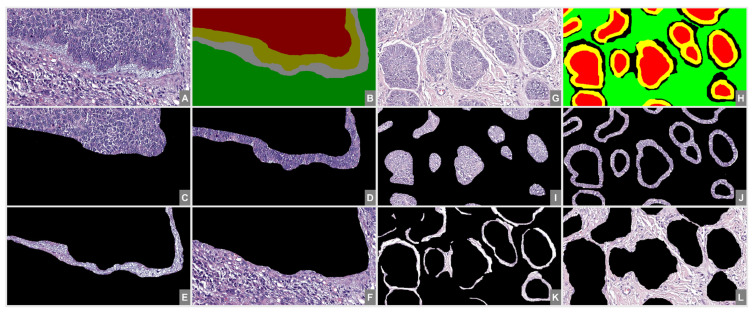
Proposed image segmentation. From (**A**–**F**)—N subtype; from (**G**–**L**)—MN subtype; (**A**,**G**)—original image; (**B**,**H**)—segmentation mask; (**C**,**I**)—T; (**D**,**J**)—TT; (**E**,**K**)—PC; (**F**,**L**)—S.

Each image was manually segmented by a trained pathologist with regard to the four components introduced in the first section (T, TT, PC, S). Using the segmentation mask, four different images were generated that contained only the selected component’s pixels. A sample of two-subtype segmentation, with the resulting segmented images, is presented in Figure 1.

After preparing the dataset, the analysis was split into two different components: (1) a classical morphometric approach with Haralick texture features and histogram moments and (2) a semantic segmentation approach with deep learning. Both approaches aimed to quantify whether there were any textural differences between the two subtypes.

#### 2.2.2. Morphometric Analysis

A common approach for texture analysis is to use the gray-level co-occurrence matrix (GLCM).

The GLCM counts how many times the value *i* occurs horizontally adjacent to a pixel with the value *j* [71]. The offset (distance between the pixel of interest and its neighbor) was set to 1 and no symmetry was considered [71].

For the GLCM computation, the four segmented images were converted to their grayscale (8-bit) version. The number of levels in GLCM was empirically set to 9. Due to the fact that the background was represented as black in the segmented images, the first line and row of the resulting GLCM were removed, thus obtaining an 8 × 8 GLC matrix.

The following Haralick [72,73] texture features were computed for each image of the segmented dataset: angular second moment (energy), contrast, correlation, variance, inverse difference moment (homogeneity), sum average, sum variance, sum entropy, entropy, difference variance, difference entropy, information measure of correlation I, information measure of correlation II and maximal correlation coefficient.

The resulting values were grouped by image segment and BCC subtype. Using Student’s *t*-test, the (statistically) different values of each segment were compared and are presented in Table 1.

Another approach for texture analysis is using the histogram moments [74,75]. The formula for computing moments is given by

(1) $$m_{k}=\overset{n}{\underset{i=1}{\sum }}\frac{{\left(x_{i}-m\right)}^{k}}{n}$$

where *m_k_* is the computed moment value, *x_i_* is the value of pixel *i*, *m* is the average pixel value of the image and *n* is the number of pixels in the image.

Considering the histogram as a distribution, the first moment is the expected value (k = 1), the second moment is the variance (k = 2), and the third and fourth moments are the skewness (k = 3) and the kurtosis (k = 4), respectively. The variance, skewness, and kurtosis were computed for each image of the segmented dataset.

Similar to the analyzed Haralick texture features, the average moment values of each segment were compared using Student’s *t*-test.

#### 2.2.3. Semantic Segmentation Analysis

Semantic segmentation is the concept of grouping parts of an image that belong to the same object class. A semantic segmentation classifier labels all the pixels in an image, thus obtaining an image that is segmented by class. Many semantic segmentation techniques were described [76], with the best performing ones being convolution-based [16].

For our experiment, a DeepLab v3+ segmentation network [77] with weights initialized from a pre-trained ResNet-18 [78] network was used. For this, several layers from a ResNet-18 network trained on ImageNet were transferred (both architecture and weights) in the DeepLab v3+ network. Only the classification layer of the network was replaced to match the new number of output classes. The network was initialized using the MATLAB (Mathworks, Natick, MA 01760-2098, Portola Valley, CA, USA) built-in function [79].

Several of the default training parameters used for training the network were changed. Thus, the learning rate schedule (LearnRateSchedule) was set to “piecewis”, the learning rate drop period (LearnRateDropPeriod) to 10, the learning rate drop factor to 3 (LearnRateDropFactor to 0.3), the momentum (Momentum) to 0.9, the initial learning rate (InitialLearnRate) to 0.001 and the L2 regularization (L2Regularization) to 0.005. All the parameters were set with the aim to accelerate the network’s convergence without a premature convergence. The training was set to perform a maximum of 30 epochs, with a mini batch size of 4 and a validation patience of 10. Parameters left unchanged were initialized with their default (Mathworks-proposed) values.

Due to their stochastic characteristic, DL algorithms must be independently run several times with different input data and the evaluation result must be presented as a mean and SD. For this, the segmentation algorithm was run 100 times, where at each step, the dataset was split into subsamples of 70% training, 15% validation and 15% testing.

The network’s performance was assessed in terms of accuracy, intersection over union (IoU), and F1 score. Considering a binary choice and a two-class intersection, a classified object could fall in one of four classes: true positive (TP), true negative (TN), false positive (FP) and false negative (FN).

Accuracy is defined as

Accuracy = (TP + TN)/(TP + TN + FP + FN)(2)

IoU is defined as

IoU score = TP/(TP + FP + FN)(3)

In order to compute the F1 score, the precision and recall are first defined as:

Precision = TP/(TP + FP)(4)

Recall = TP/(TP + FN)(5)

Based on Equations (4) and (5), the F1 score is defined as:

F1 score = (2× Precision × Recall)/(Precision + Recall)(6)

The average results for the semantic segmentation are presented in Table 2.

The best performing network was selected and used for further assessment. Thus, the whole dataset was tested and the resulting confusion matrix is presented in Table 3.

#### 2.2.4. WSI Automatic Segmentation

**Figure 2:**
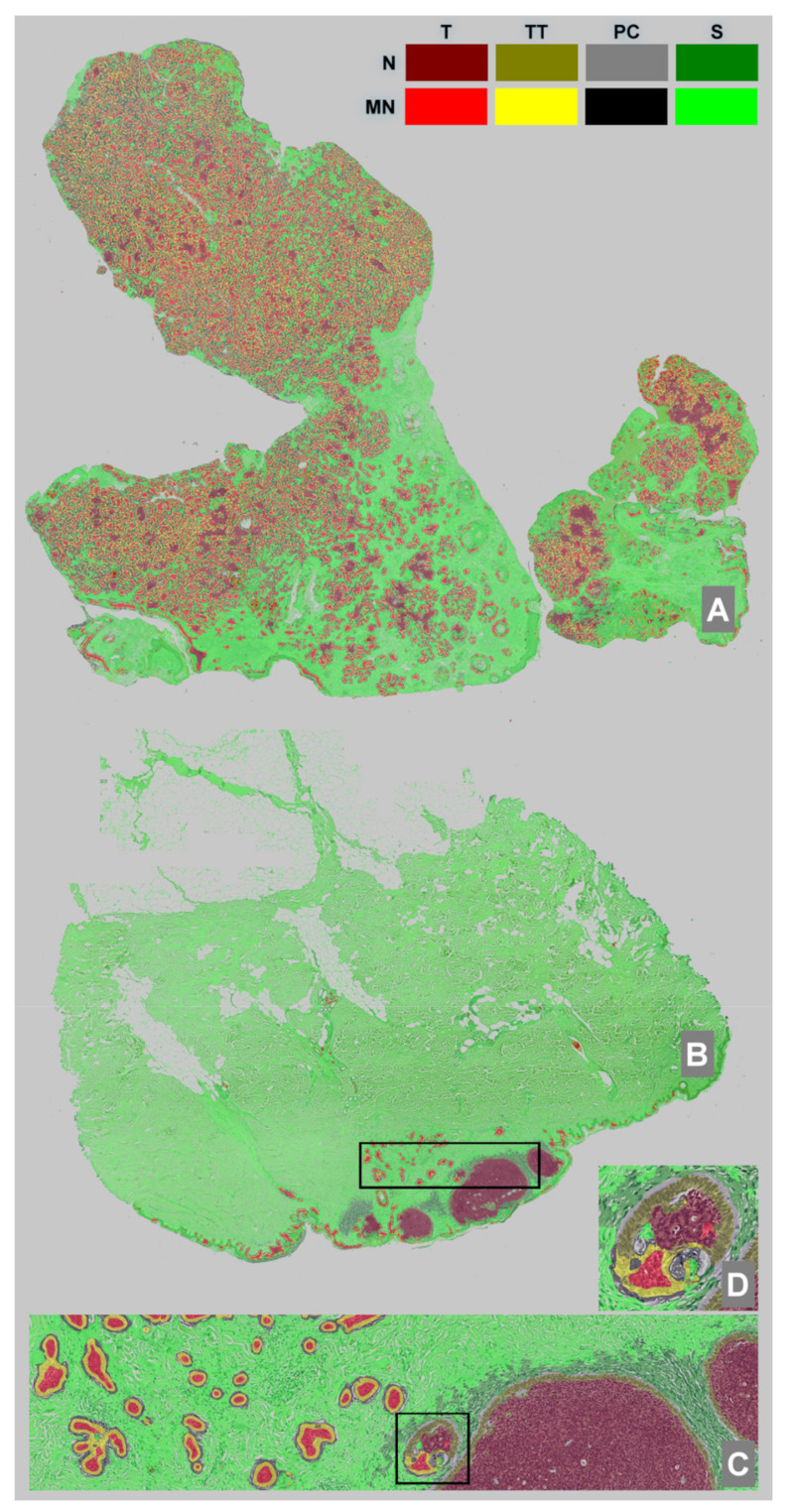
WSI segmentation using the best performing network. (**A**)—WSI with MN subtype, (**B**)—WSI with both N and MN subtypes, (**C**)—detail of the selection from (**B**,**D**)—detail of the selection from (**C**). Color labels for the segmented objects are available in the top-right corner.

Using PMA.start’s API, which is free software offered by pathomation.com (accessed on 30 May 2022) [80], WSI images were brought into the MATLAB workspace and were segmented using the best performing network obtained in Section 2.2.3. The average time for segmenting a WSI was about 6 minutes on an Intel In(R) Silver 4216 CPU @ 2.10 GHz processor with 128 GB RAM and a Quadro RTX 6000 video adapter. A sample of two segmented WSIs is present in Figure 2.

## 3. Results

The results of the morphometric analysis assessment in terms of the Haralick texture features are presented in Table 1. The more the features were significantly different between the two subtypes, the more different their texture was.

All computed histogram moments showed statistically different values for all the components (T, TT, PC, S), except for the variance (second moment) of the S component.

**Table 1:** Number of Haralick texture features with statistically different averages between subtypes.

Segment Component	Number of Statistically Different Features ^1^
T	4
TT	2
PC	12
S	5

^1^ The theoretical maximum was 14, representing all the computed texture features.

The results of the semantic segmentation assessment are presented in Table 2. As seen in Table 2, the best accuracy score was for the S-MN component, which also had the best IoU score with 0.92, while the best F1 score was for PC-N. The lowest performance regarding accuracy was for the T-N component, regarding IoU for TT-N, and regarding the F1 score for PC-MN.

Table 2 refers to the average performance of 100 network runs, while Table 3 refers to the best performing network applied to the whole dataset. The same network was used for the WSI segmentation, with an example shown in Figure 2.

**Table 2:** Semantic segmentation performance.

Segment Component	Accuracy	IoU	F1 Score
T-N	0.75	0.70	0.56
TT-N	0.79	0.47	0.63
PC-N	0.76	0.61	0.76
S-N	0.85	0.75	0.66
T-MN	0.88	0.81	0.64
TT-MN	0.83	0.34	0.39
PC-MN	0.86	0.59	0.54
S-MN	0.94	0.92	0.69
AVERAGE	0.83	0.65	0.61

Looking at the confusion matrix in Table 3, we note that the highest confusion rate for the network was between the classes T-N and TT-N, and also for the classes S-N and PC-N, while the lowest confusion was for the S-MN class.

**Table 3:** Normalized confusion matrix of the best performing semantic segmentation network on the whole dataset.

		T-N	TT-N	PC-N	S-N	T-MN	TT-MN	PC-MN	S-MN
Target Classes	T-N	0.75	0.12	0.01	0.00	0.10	0.00	0.00	0.00
	TT-N	0.09	0.79	0.08	0.00	0.00	0.03	0.00	0.00
	PC-N	0.01	0.10	0.76	0.10	0.00	0.00	0.03	0.00
	S-N	0.00	0.00	0.12	0.85	0.00	0.00	0.01	0.01
	T-MN	0.04	0.00	0.00	0.00	0.88	0.05	0.01	0.00
	TT-MN	0.00	0.01	0.00	0.00	0.06	0.83	0.09	0.00
	PC-MN	0.00	0.00	0.01	0.00	0.00	0.06	0.86	0.07
	S-MN	0.00	0.00	0.00	0.00	0.00	0.00	0.05	0.94
		Predicted Classes							

When comparing the patterns of the N and MN subtypes, we discovered that the PCs were quite different (12/14 Haralick texture features), while the TTs were rather similar (2/14 Haralick texture features). However, all the computed histogram moments showed statistically different values for the two subtypes on both the PC and TT components.

The WSI image with a zoomed-in detail focusing on a mixed zone containing both nodular and micronodular subtypes is presented in Figure 2.

## 4. Discussion

### 4.1. Results Analysis—General Remarks

After running the experiment, we ended up with two types of measurements. The first type of measurement fell into the “descriptive concept” category, as it showed how many of the Haralick texture features were different between the two BBC subtypes and the four defined classes (Table 1). The second type of measurement fell into the “inferential concept” category, as the resulting segmentation networks decided which pixel fell into which class based on the information learned in the training phase. The aim of training a segmentation network that was capable of segmenting four different components of two BCC subtypes was not to obtain an accurate segmentation (though an average accuracy of 83% is good), but to assess the inter-class performances as markers for similitude between patterns.

The classical morphometric analysis produced a lot of information. The numbers of Haralick texture features that differed for T and TT were 4/14 and 2/14, respectively. This showed a high similitude between the classes, and the fact that TT had the lowest value could indicate that the palisading had a similar function/mechanism. Twelve out of the fourteen Haralick texture features had statistically different values on the PC classes of the two subtypes. The value was more than double those found for T and TT. This could indicate that the possible difference between the output of the two malignancies could have its origin in the cleft formation. Last but not least, S had five significant different Haralick texture features (5/14). This fell in line with our expectation, as the normal tissue that is far enough from the tumor is actually similar (the same) between the subtypes, as any kind of signaling is unlikely.

The semantic segmentation analysis produced a lot more information that was partly in line with the morphometric analysis.

The first observation of the performance assessments (Table 2) showed that MN outperformed N in the matter of accuracy and IoU and had opposite results for the F1 score. This could be explained in part by a lower in-class variation of the MN subtype and a larger pixel representation for the N one.

The T component has a similar behavior for accuracy and IoU, with values above 0.7 for N and above 0.8 for MN (Table 2). This would translate to relatively high inter-class differentiation. From the confusion matrix (Table 3), we can observe that it was more likely that T was mistakenly classified as TT than that the two subtypes were misclassified with one another: (T-N vs. TT-N = 0.12) vs. (T-N vs. T-MN = 0.10) and (T-MN vs. TT-MN = 0.05) vs. (T-MN vs. T-N = 0.04).

The TT component had better accuracy in both subtypes than T but showed a lower performance in the matter of IoU (Table 2). Corroborating the information with the data from the confusion matrix (Table 3), it stands out that the TT is being misclassified as T and PC, and the confusion between the two BBC subtypes (TT-M and TT-MN) was relatively low. Nevertheless, in the matter of IoU, TT fell under T, in agreement with the classical morphological analysis (Table 1).

On one hand, the PC component had a similar behavior with the TT in regard to confusing nearby patterns in both subtypes; thus misclassifications were made with PC and S. On the other hand, inter-subtype confusion was more present, e.g., PC-N was misclassified as PC-NM 3% of the time while PC-MN was misclassified as PC-N 1% of the time (Table 3). The performance values, though smaller, were comparable with the ones from the TT, which was in opposition with the findings on the classic morphometric approach where the differences were large, i.e., 14 vs. 5 statistically different Haralick texture features.

### 4.2. Results Analysis of Tumor Stromas

The S component had the best performance for both the accuracy and IoU metrics (Table 2). Following Table 3, we observed that the common misclassification on both subtypes was with PC. For some reason, the classifier rarely mistook the S subtype; this was in opposition to classical morphometric assessment. When assessing the number of Haralick texture features with statistically different averages between the two subtypes, we found 5 out of 14 distinctive features, while the only similar moment (the variance) was on the S component, meaning that in our data set, the stromas were relatively similar between the two subtypes. However, the literature states that an intensely hyalinized stroma is associated with high-risk BCC and a fibrous stroma with low-risk BCC [45,46]. In our analysis, the distance of the surrounding stroma to the tumor was not taken in consideration; thus, normal tissue probably made up most of the analyzed area, resulting in a similar pattern [45,46]. In our research, in the analyzed images, we did not differently label the non-tumoral stroma from the tumor stroma; therefore, the software analyzed everything surrounding the tumor in the image. Given this, we do not consider it appropriate that our results should be considered for any literature comparison.

In the WSI analysis, the stromal inflammatory infiltrate between the N and MN subtype were different: we found scattered inflammatory infiltrate in the stroma closely surrounding the MN subtype, while for the N subtype, the infiltrate was abundant, but it was not as close to the tumor island as the one surrounding the MN islands. This is consistent with other studies in literature, such as the one done by Kaur et al., where they described a loss of inflammation in micronodular growth pattern (r = 0.2/0.5, *p* ≤ 0.001) and the mean inflammatory infiltrate was lower in high-risk groups but with more abundant plasma cell and lymphoid follicle formation when compared with the low-risk group [47]. On the other hand, Dunham et al. studied the immune response in various BCC subtypes and observed a dense peritumoral inflammatory infiltrate in the majority of the high-risk subtypes (including micronodular) and a mild one in the low-risk ones [40]. In regard to the type of inflammatory response, Lefrançois et al. demonstrated a difference in the type of inflammatory cells depending on the risk group, where a macrophage-rich inflammatory infiltrate was more representative of the high-risk group and a predominantly lymphocytic infiltrate was more representative of the low-risk group [41].

### 4.3. Results Analysis of Tumor Islands (Including Peripheral Palisaded Basaloid Cells)—T and TT

**Figure 3:**
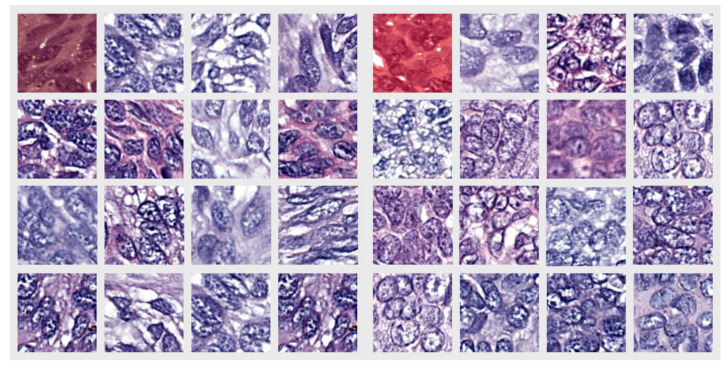
Randomly selected crops from the T component dataset, where the network uniformly segmented the area with only the correct label. The left group represents the N subtype and the right group represents the MN subtype. The first image of each group with overlaid labels represents samples from the nodule in Figure 2D.

Looking at Figure 2C, we can see the network produced a good performance when segmenting the image and that it correctly identified the two different BCC subtypes that were present on the same WSI. This led us to the idea that pathologists should also be able to find textural differences between the tumors and, in particular, within the T component. To support our theory, we selected 100 × 100-pixel patches from all the images in the dataset where the network uniformly segmented the area with only the correct label. A random sample of the resulting patches is presented in Figure 3.

Looking at the pictures in Figure 3, pattern differences between the two groups of images can be observed. In the left group (representing cells from the tumor island of the N type), the cells were more elongated and the intercellular matrix was better represented, i.e., the cells were more separated from each other. The right group, representing cells from the tumor island of the MN type, had more round or polygonal cells and the intercellular matrix appeared to be sparse. It was previously noted that in other malignancies, different intercellular differences within different patterns of the same tumor were present [81,82]. Furthermore, the elongated cells from the left group appeared to be oriented in one direction and were positioned one after another, while those from the right group had no clear orientation. These observations are important given that the images were from outside the TT zone, and thus, the influence of the surrounding stroma was improbable.

Another interesting observation can be made regarding Figure 2C,D, where a single BCC nodule was highlighted from a mixed tumor containing both N and MN subtypes. According to the definition of nodule size (an MN nodule is required to be smaller than 0.15 mm), the pathologists labeled the nodule as being part of the N subtype, but the software labeled the same nodule as part MN and part N. Without the information provided by the segmentation network, this nodule would only have been labeled as N. Going further into the details from Image 2D, we note that the network correctly predicted the TT, TS and S surrounding the islands of different types within the same nodule.

In addition to these morphological distinctions, authors such as Oh et al. also observed discrepancies in beta-catenin expression, which is increased in the micronodular subtype relative to the nodular subtype [39]. In the same study, they raised questions about a dysregulation mechanism of the beta-catenin E-cadherin complex in this MN BCC because of the nuclear location of the beta-catenin expression in this subtype [39]. Although the results were not statistically significant, beta-catenin was more expressed in the peripheral palisading portion of tumor islets than in the center of tumor islands [39]. In regard to MT1-MMP, Oh et al. found the same expression in both the peripheral palisading part of BCC tumor islets and in the central part of BCC tumor islands [39]. However, Son et al. observed that marked expression of MMP-1 in the tumor stroma also causes structural changes at the periphery of the tumor through a loss of peripheral palisading, which in turn leads to a poorly differentiated histological appearance that is correlated with a poor prognosis [83]; this finding has a particular significance in our study regarding the MN subtype.

### 4.4. Results Analysis of Peritumoral Clefts

Researchers have attempted to find an explanation of peritumoral clefts for years, and even in the 1990s, researchers such as Crowson, Sexton and Hendrix saw a difference in peritumoral clefts of the micronodular subtypes, stating that they are an uncommon finding in this subtype [68,69,70].

The texture analysis showed that the most significant differences between the two subtypes were in terms of the peritumoral cleft (Table 1). We find it important to note that the differences observed were primarily qualitative. Thus, we showed that, when present, these clefts were qualitatively different in the nodular and micronodular subtypes. Using Alcian blue stain, Ulrich et al. demonstrated the presence of mucin deposits in the PC in some subtypes of BCC, such as the nodular subtype [60]. However, in their study, they did not have cases of MN BCCs. Sahu et al. described the presence of mucin and amyloid deposits in less-aggressive nodular BCCs; however, they also did not study any MN BCCs [84]. Another hypothesis is that epithelial membrane breakdown causes peritumoral retraction, with laminin-5 perhaps playing a role [62,63]. On the other hand, other researchers disagree [64]. Newer studies, such as the one by Mentzel et al., showed that the extracellular matrix breakdown that happens during tumor growth is the source of such clefts [61].

In regard to quantity, out of the 201 MN images, 191 showed partial, focal or all-around peritumoral clefts. Although as previously stated, even though PCs are a common characteristic of BCC [6,57], they are usually uncommon in MN BCC [68,70]. A possible explanation for their abundance in our dataset was the fact that the majority of micronodular images from our cases were obtained from mixed tumors with both nodular and micronodular patterns. In our practice, we often find mixed patterns in BCC, and the most common mixed pattern we find is nodular combined with micronodular. Of course, in this situation, it is of great importance to consider tangential sectioning and irregular margins of large nodules, as researchers such as LeBoit et al. and the Australian Cancer Network also suggest [6,12]. However, the mixture of a micronodular pattern with nodular pattern is quite common, as stated in the latest edition of the WHO Classification of Skin Tumors [13].

From the confusion matrix in Table 3, we see that PC-MN was rarely confused with PC-N, but it was confused with TT-MN and S-MN. The same went for PC-N, which was confused with TT-N and S-N. This could, in part, be explained by the imperfect pathologist’s annotation used for model training, where similar pixels were classified into different classes.

### 4.5. Future Work

As we demonstrated that the two variants were different cancers with regard to their morphologies, the next step is to uncover the possible epigenetic modifications that are responsible for these differences. Laser capture microdissection (LCM) is a technology that uses a laser to cut and extract portions of tissue at a microscopic level that can be later used for further analysis. LCM can be applied to almost all fields of molecular investigation, including proteomics and transcriptomics [85]. A review of epigenetic modifications in skin [86] highlighted possible uses of LCM in skin tumors and identified some specific diseases where the results are promising, such as in melanoma [87] and cutaneous T-cell lymphoma [88], but did not identify any relevant BCC study.

Furthermore, the resulting specimens from the LCM could also be transferred to a mass analyzer [89], thus obtaining mass spectra profiles for the selected cancer samples. A step forward was made in this direction and the separation of normal vs. BCC tissue was done with high accuracy with an aim toward real-time margin assessment during BCC surgery [90].

Analyzing the two tumor variants with respect to the four components (T, TT, PC and S) taken into consideration in our study by using these new approaches could produce new information that can be used to further identify the differences.

### 4.6. Brief Summary

In order to evaluate the similarities and differences between patterns of the four components (T, TT, PC, S) amongst the two subtypes of BCC (N and MN), two measurements were used: a descriptive one (Haralick texture features and histogram moments) and an inferential one (which pixels fell into which class based on the semantic segmentation).

Tumor stroma analysis revealed that both accuracy and IoU metrics performed well for S. The Haralick texture features and histogram variance indicated that the stromas in our data set were generally similar across the two subtypes. Although studies have described differences in stromas between N and MN subtypes, since we did not distinguish between the non-tumoral and tumoral stroma, normal tissue most likely made up the majority of the examined area, resulting in a similar pattern. With this being the case, we do not believe it is proper to compare the outcomes. On the other hand, the WSI analysis showed distinct stromal inflammatory infiltrate between the two subtypes, with scattered inflammatory infiltrate in the stroma intimately enclosing the MN subtype and was abundant, although it was not as close to the tumor island inflammation in the N subtype.

The results analysis of the tumor islands (including peripheral palisaded basaloid cells) showed similarities between TT components, suggesting a similar mechanism and some dissimilarities between the T components. Randomly selected crops from the T component presented morphological differences in the cell shape, orientation and intercellular matrix between N and MN BCCs. The N subtype cells were more elongated, had a similar orientation and had a more abundant intercellular matrix when compared with the rounder and unorganized MN cells. Furthermore, the semantic segmentation network was able to highlight the MN subtype within a tumor island that was labeled as N by the pathologists (Figure 2C,D).

Out of the four analyzed components, the most significant difference between the morphology of the two subtypes was represented by the PC component. We found that these clefts, when present, were fundamentally distinct in the N and MN subtypes. These differences were mostly qualitative and need further study to highlight the exact origin of those morphological differences.

## 5. Conclusions

The coupling of the standard morphometric approach with Haralick texture features and histogram moments and the semantic segmentation with deep learning analysis of BCC MN and N subtypes provided new insights into the characterization of these two subtypes. PC’s pattern varied the most between the two subtypes, while the tumor cells in the palisading zone (TT) had the most similar pattern of the two groups.

We identified distinct pathological patterns of the T component in random fields of the tumor island that did not contain peripheral palisading. The N subtype had more elongated nuclei that followed the same directions and were positioned one after the other as opposed to the MN subtype, which has rounded nuclei with no visible alignment. Moreover, the intercellular matrix was more abundant in the N subtype T component as opposed to the MN subtype.

Deep learning techniques brought new insight into the morphologies of nodular and micronodular subtypes of BCC.

## Data Availability

Data used in the present study can be shared upon reasonable request.

## References

[1] Lomas A., Leonardi-Bee J., Bath-Hextall F. (2012). A Systematic Review of Worldwide Incidence of Nonmelanoma Skin Cancer. Br. J. Dermatol..

[2] Rubin A.I., Chen E.H., Ratner D. (2005). Basal-Cell Carcinoma. N. Engl. J. Med..

[3] Peris K., Fargnoli M.C., Garbe C., Kaufmann R., Bastholt L., Seguin N.B., Bataille V., del Marmol V., Dummer R., Harwood C.A. (2019). Diagnosis and Treatment of Basal Cell Carcinoma: European Consensusebased Interdisciplinary Guidelines. Eur. J. Cancer.

[4] Trakatelli M., Morton C., Nagore E., Ulrich C., del Marmol V., Peris K., Basset-Seguin N. (2019). Update of the European Guidelines for Basal Cell Carcinoma Management. Eur. J. Dermatol..

[5] Marzuka A.G., Book S.E. (2015). Basal Cell Carcinoma: Pathogenesis, Epidemiology, Clinical Features, Diagnosis, Histopathology, and Management. Yale J. Biol. Med..

[6] Elder D.E., Massi D., Scolyer R.W.R. (2018). WHO Classification of Skin Tumours. Skin Tumours. Pathology and Genetics.

[7] McKenzie C.A., Chen A.C., Choy B., Fernández-Peñas P., Damian D.L., Scolyer R.A. (2016). Classification of High Risk Basal Cell Carcinoma Subtypes: Experience of the ONTRAC Study with Proposed Definitions and Guidelines for Pathological Reporting. Pathology.

[8] Nedved D., Tonkovic-Capin V., Hunt E., Zaidi N., Kucenic M.J., Graves J.J., Fraga G.R. (2014). Diagnostic Concordance Rates in the Subtyping of Basal Cell Carcinoma by Different Dermatopathologists. J. Cutan. Pathol..

[9] Cohen P.R., Schulze K.E., Nelson B.R. (2006). Basal Cell Carcinoma with Mixed Histology: A Possible Pathogenesis for Recurrent Skin Cancer. Derm. Surg..

[10] Nilsson K.D., Neittaanmäki N., Zaar O., Angerer T.B., Paoli J., Fletcher J.S. (2020). TOF-SIMS Imaging Reveals Tumor Heterogeneity and Inflammatory Response Markers in the Microenvironment of Basal Cell Carcinoma. Biointerphases.

[11] IARC Publications Website—Pathology and Genetics of Skin Tumours. https://publications.iarc.fr/Book-And-Report-Series/Who-Classification-Of-Tumours/Pathology-And-Genetics-Of-Skin-Tumours-2005.

[12] Clinical Practice Guide: Basal Cell Carcinoma, Squamous Cell Carcinoma (and Related Lesions): A Gu|National Library of Australia. https://catalogue.nla.gov.au/Record/4580606.

[13] Scolyer R.A., Elder D.E., Massi D., Scolyer R.A., Willemze R. (2018). Keratinocytic/Epidermal Tumours. WHO Classification of Skin Tumours.

[14] Litjens G., Kooi T., Bejnordi B.E., Setio A.A.A., Ciompi F., Ghafoorian M., van der Laak J.A.W.M., van Ginneken B., Sánchez C.I. (2017). A Survey on Deep Learning in Medical Image Analysis. Med. Image. Anal..

[15] Chen L.C., Papandreou G., Kokkinos I., Murphy K., Yuille A.L. (2018). DeepLab: Semantic Image Segmentation with Deep Convolutional Nets, Atrous Convolution, and Fully Connected CRFs. IEEE Trans. Pattern Anal. Mach. Intell..

[16] Minaee S., Boykov Y.Y., Porikli F., Plaza A.J., Kehtarnavaz N., Terzopoulos D. (2020). Image Segmentation Using Deep Learning: A Survey. IEEE Trans. Pattern Anal. Mach. Intell..

[17] Echle A., Rindtorff N.T., Brinker T.J., Luedde T., Pearson A.T., Kather J.N. (2020). Deep Learning in Cancer Pathology: A New Generation of Clinical Biomarkers. Br. J. Cancer.

[18] Retamero J.A., Aneiros-Fernandez J., del Moral R.G. (2020). Complete Digital Pathology for Routine Histopathology Diagnosis in a Multicenter Hospital Network. Arch. Pathol. Lab. Med..

[19] Steiner D.F., Macdonald R., Liu Y., Truszkowski P., Hipp J.D., Gammage C., Thng F., Peng L., Stumpe M.C. (2018). Impact of Deep Learning Assistance on the Histopathologic Review of Lymph Nodes for Metastatic Breast Cancer. Am. J. Surg. Pathol..

[20] van Zon M.C.M., van der Waa J.D., Veta M., Krekels G.A.M. (2021). Whole-Slide Margin Control through Deep Learning in Mohs Micrographic Surgery for Basal Cell Carcinoma. Exp. Derm..

[21] Campanella G., Hanna M.G., Geneslaw L., Miraflor A., Werneck Krauss Silva V., Busam K.J., Brogi E., Reuter V.E., Klimstra D.S., Fuchs T.J. (2019). Clinical-Grade Computational Pathology Using Weakly Supervised Deep Learning on Whole Slide Images. Nat. Med..

[22] Jiang Y.Q., Xiong J.H., Li H.Y., Yang X.H., Yu W.T., Gao M., Zhao X., Ma Y.P., Zhang W., Guan Y.F. (2020). Recognizing Basal Cell Carcinoma on Smartphone-Captured Digital Histopathology Images with a Deep Neural Network. Br. J. Derm..

[23] Kimeswenger S., Tschandl P., Noack P., Hofmarcher M., Rumetshofer E., Kindermann H., Silye R., Hochreiter S., Kaltenbrunner M., Guenova E. (2021). Artificial Neural Networks and Pathologists Recognize Basal Cell Carcinomas Based on Different Histological Patterns. Mod. Pathol..

[24] Bungărdean R.M., Şerbănescu M.-S., Streba C.T., Crişan M. (2021). Deep Learning with Transfer Learning in Pathology. Case Study: Classification of Basal Cell Carcinoma. Rom. J. Morphol. Embryol..

[25] Leef G., Thomas S.M. (2013). Molecular Communication between Tumor-Associated Fibroblasts and Head and Neck Squamous Cell Carcinoma. Oral Oncol..

[26] Bertheim U., Hofer P.Å., Engström-Laurent A., Hellström S. (2004). The Stromal Reaction in Basal Cell Carcinomas. A Prerequisite for Tumour Progression and Treatment Strategy. Br. J. Plast. Surg..

[27] Moy R.L., Potter T.S., Uitto J. (2000). Increased Glycosaminoglycans Production in Sclerosing Basal Cell Carcinoma-Derived Fibroblasts and Stimulation of Normal Skin Fibroblast Glycosaminoglycans Production by a Cytokine-Derived from Sclerosing Basal Cell Carcinoma. Derm. Surg..

[28] Lesack K., Naugler C. (2012). Morphometric Characteristics of Basal Cell Carcinoma Peritumoral Stroma Varies among Basal Cell Carcinoma Subtypes. BMC Derm..

[29] Reuter J.A., Ortiz-Urda S., Kretz M., Garcia J., Scholl F.A., Pasmooij A.M.G., Cassarino D., Chang H.Y., Khavari P.A. (2009). Modeling Inducible Human Tissue Neoplasia Identifies an Extracellular Matrix Interaction Network Involved in Cancer Progression. Cancer Cell.

[30] Parrott J.A., Nilsson E., Mosher R., Magrane G., Albertson D., Pinkel D., Gray J.W., Skinner M.K. (2001). Stromal-Epithelial Interactions in the Progression of Ovarian Cancer: Influence and Source of Tumor Stromal Cells. Mol. Cell Endocrinol..

[31] Mantovani A. (2011). B Cells and Macrophages in Cancer: Yin and Yang. Nat. Med..

[32] Kaporis H.G., Guttman-Yassky E., Lowes M.A., Haider A.S., Fuentes-Duculan J., Darabi K., Whynot-Ertelt J., Khatcherian A., Cardinale I., Novitskaya I. (2007). Human Basal Cell Carcinoma Is Associated with Foxp3+ T Cells in a Th2 Dominant Microenvironment. J. Investig. Derm..

[33] Kalluri R., Zeisberg M. (2006). Fibroblasts in Cancer. Nat. Rev. Cancer.

[34] Orimo A., Gupta P.B., Sgroi D.C., Arenzana-Seisdedos F., Delaunay T., Naeem R., Carey V.J., Richardson A.L., Weinberg R.A. (2005). Stromal Fibroblasts Present in Invasive Human Breast Carcinomas Promote Tumor Growth and Angiogenesis through Elevated SDF-1/CXCL12 Secretion. Cell.

[35] Al-Rakan M.A., Colak D., Hendrayani S.F., Al-Bakheet A., Al-Mohanna F.H., Kaya N., Al-Malik O., Aboussekhra A. (2013). Breast Stromal Fibroblasts from Histologically Normal Surgical Margins Are Pro-Carcinogenic. J. Pathol..

[36] Omland S.H., Wettergren E.E., Mourier T., Hansen A.J., Asplund M., Mollerup S., Robert R. (2017). Cancer Associated Fibroblasts (CAFs) Are Activated in Cutaneous Basal Cell Carcinoma and in the Peritumoural Skin. BMC Cancer.

[37] Takahashi M., Tsunoda T., Seiki M., Nakamura Y., Furukawa Y. (2002). Identification of Membrane-Type Matrix Metalloproteinase-1 as a Target of the Beta-Catenin/Tcf4 Complex in Human Colorectal Cancers. Oncogene.

[38] Liu P., Yang J., Pei J., Pei D., Wilson M.J. (2010). Regulation of MT1-MMP Activity by β-Catenin in MDCK Non-Cancer and HT1080 Cancer Cells. J. Cell Physiol..

[39] Oh S.T., Kim H.S., Yoo N.J., Lee W.S., Cho B.K., Reichrath J. (2011). Increased Immunoreactivity of Membrane Type-1 Matrix Metalloproteinase (MT1-MMP) and β-Catenin in High-Risk Basal Cell Carcinoma. Br. J. Derm..

[40] Duman N., Korkmaz N.Ş., Erol Z. (2016). Host Immune Responses and Peritumoral Stromal Reactions in Different Basal Cell Carcinoma Subtypes: Histopathological Comparison of Basosquamous Carcinoma and High-Risk and Low-Risk Basal Cell Carcinoma Subtypes. Turk. J. Med. Sci..

[41] Lefrançois P., Xie P., Gunn S., Gantchev J., Villarreal A.M., Sasseville D., Litvinov I.V. (2020). In Silico Analyses of the Tumor Microenvironment Highlight Tumoral Inflammation, a Th2 Cytokine Shift and a Mesenchymal Stem Cell-like Phenotype in Advanced in Basal Cell Carcinomas. J. Cell Commun. Signal..

[42] Tlsty T.D., Coussens L.M. (2006). Tumor Stroma and Regulation of Cancer Development. Annu. Rev. Pathol..

[43] Christian M.M., Moy R.L., Wagner R.F., Yen-Moore A. (2001). A Correlation of Alpha-Smooth Muscle Actin and Invasion in Micronodular Basal Cell Carcinoma. Derm. Surg..

[44] Etienne-Manneville S. (2004). Actin and Microtubules in Cell Motility: Which One Is in Control?. Traffic.

[45] Dixon A.Y., Lee S.H., McGregor D.H. (1989). Factors Predictive of Recurrence of Basal Cell Carcinoma. Am. J. Derm..

[46] Jacobs G.H., Rippey J.J., Altini M. (1982). Prediction of Aggressive Behavior in Basal Cell Carcinoma. Cancer.

[47] Kaur P., Mulvaney M., Andrew Carlson J. (2006). Basal Cell Carcinoma Progression Correlates with Host Immune Response and Stromal Alterations: A Histologic Analysis. Am. J. Derm..

[48] Asplund A., Sivertsson Å., Bäckvall H., Ahmadian A., Lundeberg J., Ponten F. (2005). Genetic Mosaicism in Basal Cell Carcinoma. Exp. Derm..

[49] Egeblad M., Werb Z. (2002). New Functions for the Matrix Metalloproteinases in Cancer Progression. Nat. Rev. Cancer.

[50] Philips N., Auler S., Hugo R., Gonzalez S. (2011). Beneficial Regulation of Matrix Metalloproteinases for Skin Health. Enzym. Res..

[51] Strongin A.Y. (2006). Mislocalization and Unconventional Functions of Cellular MMPs in Cancer. Cancer Metastasis Rev..

[52] Tian X., Liu Z., Niu B., Zhang J., Tan T.K., Lee S.R., Zhao Y., Harris D.C.H., Zheng G. (2011). E-Cadherin/β-Catenin Complex and the Epithelial Barrier. J. Biomed. Biotechnol..

[53] El-Bahrawy M., El-Masry N., Alison M., Poulsom R., Fallowfield M. (2003). Expression of Beta-Catenin in Basal Cell Carcinoma. Br. J. Derm..

[54] Wong C.S.M., Strange R.C., Lear J.T. (2003). Basal Cell Carcinoma. BMJ.

[55] Kim D.P., Kus K.J.B., Ruiz E. (2019). Basal Cell Carcinoma Review. Hematol. Oncol. Clin. North Am..

[56] Ramdial P.K., Madaree A., Reddy R., Chetty R. (2000). Bcl-2 Protein Expression in Aggressive and Non-Aggressive Basal Cell Carcinomas. J. Cutan. Pathol..

[57] Ríos-Martín J.J., Moreno-Ramírez D., González-Cámpora R. (2012). What Is the Cause of Retraction Spaces Associated with Basal Cell Carcinoma?. J. Cutan. Pathol..

[58] Hansen T., Nardone B. (2013). Characterizing Peritumoral Clefts in Basal Cell Carcinoma with Histologic Staining and Reflectance Confocal Microscopy. J. Am. Acad. Derm..

[59] Ghita M.A., Caruntu C., Rosca A.E., Kaleshi H., Caruntu A., Moraru L., Docea A.O., Zurac S., Boda D., Neagu M. (2016). Reflectance Confocal Microscopy and Dermoscopy for in Vivo, Non-Invasive Skin Imaging of Superficial Basal Cell Carcinoma. Oncol. Lett..

[60] Ulrich M., Roewert-Huber J., González S., Rius-Diaz F., Stockfleth E., Kanitakis J. (2011). Peritumoral Clefting in Basal Cell Carcinoma: Correlation of in Vivo Reflectance Confocal Microscopy and Routine Histology. J. Cutan. Pathol..

[61] Mentzel J., Anderegg U., Paasch U., Simon J.C., Grupp M., Grunewald S. (2022). “Retraction Artefacts” in Basal Cell Carcinomas Do Not Result from Fixation but Likely Arise by Degradation of Extracellular Matrix during Tumour Growth. J. Eur. Acad. Derm. Venereol..

[62] Bahadoran P., Perrin C., Aberdam D., Spadafora-Pisani A., Meneguzzi G., Ortonne J.-P. (1997). Altered Expression of the Hemidesmosome-Anchoring Filament Complex Proteins in Basal Cell Carcinoma: Possible Role in the Origin of Peritumoral Lacunae. Br. J. Dermatol..

[63] Drewniok C., Wienrich B.G., Schön M., Ulrich J., Zen Q., Telen M.J., Hartig R.J., Wieland I., Gollnick H., Schön M.P. (2004). Molecular Interactions of B-CAM (Basal-Cell Adhesion Molecule) and Laminin in Epithelial Skin Cancer. Arch. Derm. Res..

[64] Mostafa W.Z., Mahfouz S.M., Bosseila M., Sobhi R.M., El-Nabarawy E. (2010). An Immunohistochemical Study of Laminin in Basal Cell Carcinoma. J. Cutan. Pathol..

[65] Manola I., Mataic A., Drvar D.L., Pezelj I., Dzombeta T.R., Kruslin B. (2020). Peritumoral Clefting and Expression of MMP-2 and MMP-9 in Basal Cell Carcinoma of the Skin. In Vivo.

[66] Undén A.B., Sandstedt B., Bruce K., Hedblad M.A., Ståhle-Bäckdahl M. (1996). Stromelysin-3 MRNA Associated with Myofibroblasts Is Overexpressed in Aggressive Basal Cell Carcinoma and in Dermatofibroma but Not in Dermatofibrosarcoma. J. Investig. Derm..

[67] Poswar F.O., Fraga C.A.C., Farias L.C., Feltenberger J.D., Cruz V.P.D., Santos S.H.S., Silveira C.M., de Paula A.M.B., Guimarães A.L.S. (2013). Immunohistochemical Analysis of TIMP-3 and MMP-9 in Actinic Keratosis, Squamous Cell Carcinoma of the Skin, and Basal Cell Carcinoma. Pathol. Res. Pract..

[68] Crowson A.N., Magro C.M., Kadin M.E., Stranc M. (1996). Differential Expression of the Bcl-2 Oncogene in Human Basal Cell Carcinoma. Hum. Pathol..

[69] Sexton M., Jones D.B., Maloney M.E. (1990). Histologic Pattern Analysis of Basal Cell Carcinoma. Study of a Series of 1039 Consecutive Neoplasms. J. Am. Acad. Derm..

[70] Hendrix J.D., Parlette H.L. (1996). Micronodular Basal Cell Carcinoma. A Deceptive Histologic Subtype with Frequent Clinically Undetected Tumor Extension. Arch. Derm..

[71] Co-Occurrence Matrix—Wikipedia. https://en.wikipedia.org/wiki/Co-occurrence_matrix.

[72] HaralickTextureFeatures—File Exchange—MATLAB Central. https://www.mathworks.com/matlabcentral/fileexchange/58769-haralicktexturefeatures.

[73] Haralick R.M., Dinstein I., Shanmugam K. (1973). Textural Features for Image Classification. IEEE Trans. Syst. Man Cybern..

[74] Moment (Mathematics)—Wikipedia. https://en.wikipedia.org/wiki/Moment_(mathematics).

[75] Tan Y.Y., Sim K.S., Tso C.P. (2007). A Study on Central Moments of the Histograms from Scanning Electron Microscope Charging Images. Scanning.

[76] Thoma M. (2016). A Survey of Semantic Segmentation. arXiv.

[77] Chen L.C., Zhu Y., Papandreou G., Schroff F., Adam H. (2018). Encoder-Decoder with Atrous Separable Convolution for Semantic Image Segmentation. arXiv.

[78] He K., Zhang X., Ren S., Sun J. Deep Residual Learning for Image Recognition. Proceedings of the 2016 IEEE Conference on Computer Vision and Pattern Recognition (CVPR).

[79] Create DeepLab V3+ Convolutional Neural Network for Semantic Image Segmentation—MATLAB Deeplabv3plusLayers. https://www.mathworks.com/help/vision/ref/deeplabv3pluslayers.html.

[80] PMA.Start—Pathomation. https://www.pathomation.com/pma-start/.

[81] Pleşea I.E., Stoiculescu A., Şerbǎnescu M., Alexandru D.O., Man M., Pop O.T., Pleşea R.M. (2013). Correlations between Intratumoral Vascular Network and Tumoral Architecture in Prostatic Adenocarcinoma. Rom. J. Morphol. Embryol..

[82] Pleșea R.M., Șerbănescu M.S., Alexandru D.O., Ciovică V., Stoiculescu A., Pop O.T., Simionescu C., Pleșea I.E. (2015). Correlations Between Intratumoral Interstitial Fibrillary Network and Vascular Network in Gleason Patterns of Prostate Adenocarcinoma. Curr. Health Sci. J..

[83] Kyung D.S., Kim T.J., Youn S.L., Gyeong S.P., Ki T.H., Jin S.L., Chang S.K. (2008). Comparative Analysis of Immunohistochemical Markers with Invasiveness and Histologic Differentiation in Squamous Cell Carcinoma and Basal Cell Carcinoma of the Skin. J. Surg. Oncol..

[84] Sahu A., Cordova M., Gill M., Alessi-Fox C., Navarrete-Dechent C., González S., Iftimia N., Rajadhyaksha M., Marghoob A.A., Chen C.S.J. (2020). In Vivo Identification of Amyloid and Mucin in Basal Cell Carcinoma with Combined Reflectance Confocal Microscopy-Optical Coherence Tomography Device and Direct Histopathologic Correlation. J. Am. Acad. Derm..

[85] von Eggeling F., Hoffmann F. (2020). Microdissection-An Essential Prerequisite for Spatial Cancer Omics. Proteomics.

[86] Bhamidipati T., Sinha M., Sen C.K., Singh K. (2022). Laser Capture Microdissection in the Spatial Analysis of Epigenetic Modifications in Skin: A Comprehensive Review. Oxid. Med. Cell Longev..

[87] Sigalotti L., Fratta E., Parisi G., Coral S., Maio M. (2014). Epigenetic Markers of Prognosis in Melanoma. Methods Mol. Biol..

[88] Wu J., Salva K.A., Stutz N., Longley B.J., Spiegelman V.S., Wood G.S. (2014). Quantitative Gene Analysis of Methylation and Expression (Q-GAME) in Fresh or Fixed Cells and Tissues. Exp. Derm..

[89] Zhang H., Zhao L., Jiang J., Zheng J., Yang L., Li Y., Zhou J., Liu T., Xu J., Lou W. (2022). Multiplexed Nanomaterial-Assisted Laser Desorption/Ionization for Pan-Cancer Diagnosis and Classification. Nat. Commun..

[90] Akbarifar F., Jamzad A., Santilli A., Kauffman M., Janssen N., Connolly L., Ren K.Y.M., Vanderbeck K., Wang A., Mckay D. (2021). Graph-Based Analysis of Mass Spectrometry Data for Tissue Characterization with Application in Basal Cell Carcinoma Surgery. SPIE.

